# Efficient Face Recognition System for Operating in Unconstrained Environments

**DOI:** 10.3390/jimaging7090161

**Published:** 2021-08-26

**Authors:** Alejandra Sarahi Sanchez-Moreno, Jesus Olivares-Mercado, Aldo Hernandez-Suarez, Karina Toscano-Medina, Gabriel Sanchez-Perez, Gibran Benitez-Garcia

**Affiliations:** 1 Sección de Estudios de Posgrado e Investigación, Instituto Politécnico Nacional, Av. Santa Ana 1000, San Francisco Culhuacan, Mexico City 04440, Mexico; asanchezm1800@alumno.ipn.mx (A.S.S.-M.); jolivares@ipn.mx (J.O.-M.); alhernandezsu@ipn.mx (A.H.-S.); ltoscano@ipn.mx (K.T.-M.); gasanchezp@ipn.mx (G.S.-P.); 2 Graduate School of Informatics and Engineering, The University of Electro-Communications, Chofu-shi 182-8585, Japan

**Keywords:** real-time systems, deep neural network, computer vision, image processing

## Abstract

Facial recognition is fundamental for a wide variety of security systems operating in real-time applications. Recently, several deep neural networks algorithms have been developed to achieve state-of-the-art performance on this task. The present work was conceived due to the need for an efficient and low-cost processing system, so a real-time facial recognition system was proposed using a combination of deep learning algorithms like FaceNet and some traditional classifiers like SVM, KNN, and RF using moderate hardware to operate in an unconstrained environment. Generally, a facial recognition system involves two main tasks: face detection and recognition. The proposed scheme uses the YOLO-Face method for the face detection task which is a high-speed real-time detector based on YOLOv3, while, for the recognition stage, a combination of FaceNet with a supervised learning algorithm, such as the support vector machine (SVM), is proposed for classification. Extensive experiments on unconstrained datasets demonstrate that YOLO-Face provides better performance when the face under an analysis presents partial occlusion and pose variations; besides that, it can detect small faces. The face detector was able to achieve an accuracy of over 89.6% using the Honda/UCSD dataset which runs at 26 FPS with darknet-53 to VGA-resolution images for classification tasks. The experimental results have demonstrated that the FaceNet+SVM model was able to achieve an accuracy of 99.7% using the LFW dataset. On the same dataset, FaceNet+KNN and FaceNet+RF achieve 99.5% and 85.1%, respectively; on the other hand, the FaceNet was able to achieve 99.6%. Finally, the proposed system provides a recognition accuracy of 99.1% and 49 ms runtime when both the face detection and classifications stages operate together.

## 1. Introduction

All people commonly have the ability to instantly recognize many objects in an automatic manner because the brain is capable of performing millions of extraordinarily complex operations to perform high quality pattern recognition tasks. To allow computers to be able to emulate some pattern recognition tasks performed by the human brain, several efficient algorithms have been proposed during the last few decades to operate in constrained as well as unconstrained environments, such as video sequences [1,2,3,4]. These efforts began with the work of McCulloch and Pitts, which established the principles of understanding neuronal activity and the brain operation [5].

Face recognition is a classic problem that has received significant attention in recent years. Nevertheless, face recognition accuracy is influenced by the environment in which it is used. Particularly in unconstrained face recognition, numerous factors affect the accuracy; for example, the face images present different variations such as: pose variation, scale variation, partial occlusion, and complex illumination, resulting in low recognition accuracies. Because of the success of the deep learning architecture [6], several fields of artificial intelligence have developed deep learning based solutions, such as face recognition. Recognition methods based on deep neural networks have shown many advantages in terms of learning ability, high variability, and generalization. However, efficient algorithms still present several limitations when real-time operation is required, as well as in an unconstrained environments because it requires achieving high accuracy and computational efficiency. Therefore, face recognition still represents an important challenge in real-time applications, and it is an active research field in the context of computer vision, deep learning, real-time systems, etc.

The complexity of face recognition systems depends on the interaction of several less complex sub-systems jointly operating to solve more complex tasks; in particular, we can generalize two fundamental operations involved in the facial recognition tasks: face detection and face recognition. A face recognition system is limited in minor conditions and required to detect faces in images (or videos) regardless of the facial object appearance. Secondly, face images are then processed; subsequently, face features are extracted with a feature extractor. Finally, the system compares the extracted features with the enrolled faces to make face matching. This is why faster algorithms can greatly benefit the system performance and achieve high recognition rates. Recently, to speed up the face detection stage, some deep learning-based algorithms employ one-stage detectors such as YOLO (You Only Look Once: Unified, Real-Time Object Detection) [7] and SSD (Single Shot MultiBox Detector) [8]. Among them, the YOLO-Face method, which is one of the more efficient schemes, was developed to solve the detection problem of varying scales in face detection methods. The YOLO-Face algorithm, based on YOLOv3 [9], uses multiple levels of features maps, like the FPN (Feature Pyramid Network) [10].

This paper proposes a face recognition system for operating in an unconstrained environment which uses a modified YOLO-Face method in cascade with a classification scheme which allows for increasing the detection accuracy and reducing the computation time. We also propose an architecture combining Convolutional Neural Network (CNN) and a supervised learning algorithm to be used for classification. Many deep learning architectures use the softmax loss (and its variations) as the classifier in the fully connected layer [4]. However, several studies suggest that using other classifiers such as support vector machine (SVM) instead of the softmax function may improve the face recognition performance [11,12,13,14,15].

The main contributions of this paper are three-fold:

- A comprehensive review of some state-of-the-art real-time face detection and face recognition methods.
- A modular automatic facial recognition system based on a proposed model for facial matching using CNN features with a handcrafted supervised learning classifier, such as SVM. Note that the modularity of our proposal enables to change specific algorithms to achieve efficient performance on GPU as well as on CPU-only machines.
- A systematic evaluation of proposed and conventional methods on six publicly available datasets for facial recognition systems.

The rest of this paper is organized as follows: Section 2 describes the related work, Section 3 describes the proposed system and all the stages from the face detection to face recognition, Section 4 presents the experimental results, and, finally, Section 5 concludes this work.

## 2. Related Work

The proposed face recognition system is intended for operating in unconstrained environments, and it requires one stage for face detection, whose output is inserted into a face recognition stage. Thus, it is necessary to determine the most suitable stages and the required modifications to achieve the desired face recognition results even in highly adverse conditions. The next sections present the analysis of face detection and recognition schemes, together with the required improvements.

### 2.1. Face Detection Methods

Among automatic object detection, face detection schemes have been widely analyzed, due to their potential use, in many practical systems such as: video surveillance, biometrics, and access control systems among others, which are robustly dependent on an accurate face detection. The face is a biometric human characteristic containing important information about the person’s identity. Thus, an accurate face detection is the first stage in many applications such as face recognition, face image retrieval, facial tracking, video surveillance, etc. There are two approaches used for face detection that will be analyzed: the handcrafted based-face detectors and the deep neural networks-based face detectors.

#### 2.1.1. Handcrafted Face Detection Methods

Handcrafted face detection methods have been used since the beginning of machine learning in several computer vision applications. A wide variety of state-of-the-art algorithms for face detection have been developed in the last several years, such as, Viola–Jones (VJ) [16], the Histograms of Oriented Gradients (HOG) [17], the Local Binary Patterns (LBP) [18], and several others. The design of handcrafted features often involves finding the right trade-off between accuracy and computational efficiency. The VJ algorithm has demonstrated high performance in face detection tasks [16,19,20]. The HOG and LBP are texture-based descriptors used to find patterns in a region of interest. These two high-performance texture models have shown excellent results as feature extraction algorithms for face detection [20,21,22].

#### 2.1.2. Deep Neural Network Face Detection Methods

Recently, neural networks have been a solution for the face detection task. Several studies show that the features learned using deep neural networks in most cases provide a better generalization than the handcrafted features [23] because CNN uses deep learning approaches which are ideal for image and video processing. Thus, several successful systems that have been developed in the last few years have embraced deep neural networks for face detection [24,25,26,27,28,29,30,31]. The face detection systems based on deep neural networks can be divided into one-stage and two-stages approaches, as depicted as follows:

One-stage approaches. These schemes use a single feed-forward convolutional network architecture to directly predict classes and anchor boxes without requiring for a second stage for the proposal operation. This approach removes the candidate windows obtaining, processing, and directly classifying and regressing the candidate anchor boxes. Although this type of architecture was recently proposed, its approach allows for solving the speed problem. YOLO [7] and SSD [8] are the first one-stage detectors based on CNN. Despite the two-stage models being accurate, many of them are too slow, which limits their use in real-time applications. Thus, the one-stage methods are much more efficient for real-time face detection tasks.

Two-stage approaches. These schemes divide the face detection task into two steps: (a) obtaining candidate windows and (b) classifying and regressing the candidate windows. These approaches in a first stage generate regions of interest with the use a Region Proposal Network and, in a second stage, they send the region proposals throughout the process for object classification and bounding box regression. Commonly, the methods based on this approach achieve a higher accuracy rate. The most representative two-stage face detectors are based on R-CNN [32] collections, such as fast R-CNN [33], faster R-CNN [34], and R-FCN [35].

### 2.2. Face Recognition Methods

In recent years, deep learning based and handcrafted based feature extraction approaches have gained increasing importance in the face recognition task [4,36]. Handcrafted solutions obtain facial features from preselected primary features. In contrast, learning solutions derived facial features by learning from input data relationships using functions that use deep neural networks.

#### 2.2.1. Handcrafted Face Recognition Methods

The handcrafted facial recognition methods are made up of the relationship between feature extraction and classification. Regarding the former component, an ideal descriptor algorithm for facial regions should have a larger interpersonal variance than an intrapersonal one; in that sense, the classifier’s task will be simpler. Feature extraction algorithms for facial recognition systems focus on permanent facial features such as eyebrows, eyes, nose, mouth, or face shape. Some of these most representative descriptors are LBP [37], Gabor [38], SIFT [39], and HOG [40].

In the second component, a classifier is used to obtain the distance of the face features to differentiate among several individuals. Some of the most widely used algorithms in facial recognition tasks are Support Vector Machine (SVM), K-Neighbors Neighbors (KNN), and Random Forest (RF), for their effectiveness in multiclass classification [4,41,42,43].

#### 2.2.2. Deep Neural Network Face Recognition Methods

In the last decade, research focus has shifted towards deep learning based face recognition. Typical facial recognition methods adopt CNN architectures, including a simple CNN, multi CNNs, variants of CNN, etc. [44]. In 2014, DeepFace [45] and DeepID [46] took another step towards human level performance. Inspired by these results, deep learning-based methods have been proposed and focused on the challenges for unconstrained scenarios, providing recognition with an accuracy above of 99% on the LFW dataset [47,48,49,50,51,52,53,54].

**Figure 1:**
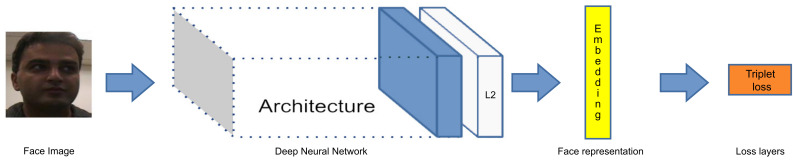
An overview of the FaceNet [48] framework.

Among the most relevant works of face recognition, the FaceNet algorithm stands out. Schroff et al. proposed this method which is based on a single deep neural network that is used for face recognition and clustering. FaceNet consists of eleven convolutional layers and three Fully Connected (FC) layers with more than 140 million (M) parameters to learn a mapping from face images. This work proposes to use triplet loss to train the model which is used to minimize the distance between an anchor and a positive sample of the same identity and to maximize the distance between the anchor and a negative sample of a different identity as pictured in Figure 1 [48]. The system was trained using between 100–200 M 2D facial images of about 8 M different identities and achieved an accuracy of 99.6% on the benchmark LFW dataset.

## 3. Proposed System

**Figure 2:**
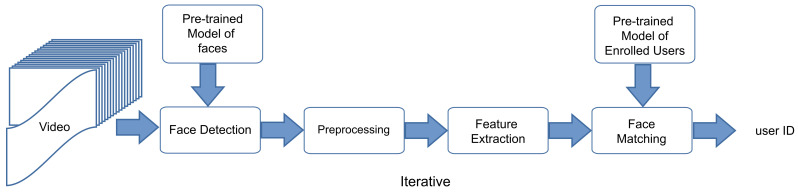
General real-time face recognition framework in video systems.

Figure 2 shows the proposed automatic face recognition system. The system consists of four stages: face detection (Section 3.1), preprocessing (Section 3.2), and feature extraction (Section 3.3). In the first stage, one or more faces are detected in the image or video frame. In the second stage, image processing techniques are used to prepare the face image for applying the machine learning model. In the third stage, the face image features are extracted. In the last stage, the facial features are compared against the known face features of enrolled users for person identification.

### 3.1. Face Detection Stage

This section presents a description of the face detection stage, which is responsible for detecting face images in the input frames on unconstrained environments. The face detector method for this stage must be able to operate with the high variability factors present in the images such as pose, scale, illumination, etc. After an extensive evaluation of several face detection scheme, the selected method is a designed for real-time applications like YOLO-Face. This method is based on a one-stage deep neural network approach. These solutions directly estimate classes and their bounding boxes without a second-stage proposal operation like faster R-CNN [34] method. The main contribution of this approach is to solve the speed problem that occurs in two-stage methods.

#### YOLO-Face

Sample availability. In this section, the main aspects of YOLO-Face scheme [31] are briefly introduced, which uses the detection network YOLOv3 [9], which is based on object detection algorithms using deep learning approaches. This scheme significantly improves the performance of face detection schemes.

Class Prediction. YOLO-Face changes the multi-label classification problem to a binary classification problem to focus on detecting only two classes: face or non-face. It minimizes the error of the model output with a multi-part loss function, inspired by the object detection network on which it is based. This loss function is composed of four parts, loss of coordinate regression, loss of confidence in anchors with objects, and loss of confidence in anchors with no objects and loss of classification. The proportion of the four parts, sub-loss is 2:1:0.5:0.5.

**Figure 3:**
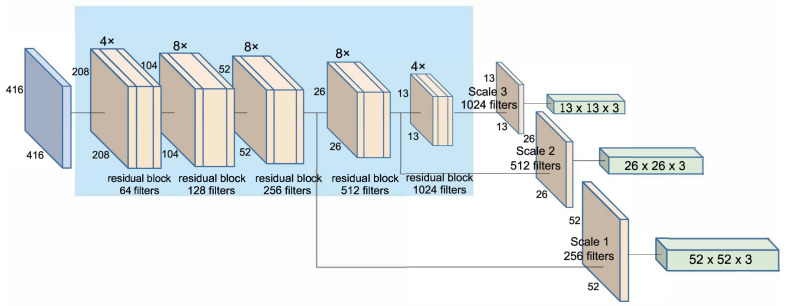
YOLO-Face (deeper darknet) architecture. The network uses different map scales to extract features, and it has a similar structure to FPN [10].

Feature extraction network. YOLO-Face uses the darknet-53 network [9], which is a hybrid approach between the *darknet-19 network* (used in YOLOv2) and that newfangled residual network stuff with 53 convolutional layers. Comparison of a YOLO-Face network with darknet-19 network shows that the first network provides better performance. It also gets better efficiency than ResNet-101 and a similar performance to ResNet-152. A problem with the YOLOv3 network is that its performance worsens when it is required to detect small-scale objects. Therefore, YOLO-Face proposes an improvement of the structure of original darknet-53 network by increasing the number of network layers of the first two residual blocks to obtain a higher quality of facial features before the characteristics of small faces on the feature map become too small. Instead of using 1× and 2× in the first two residual blocks, the number of layers increases to 4× and 8× in these blocks. This network scales down the feature maps by the convolutional layer with stride 2, which is significantly larger than YOLOv3. YOLO-Face has 71 convolutional layers called *deeper darknet*. The full network is shown in Figure 3. The results presented in [31] show that the Face Attention Network (FAN) [55] has a similar detection speed to *YOLO-Face(darknet-53)*, although the FAN is less efficient.

Prediction Across Scales. YOLO-Face performs detection on three different scales; besides that, for each scale, it predicts three bounding boxes using a similar concept to FPN [10], see Figure 3. This approach offers better performance than a multi-scale detection with the use of sizing feature maps at multiple levels. From the base feature extractor, the output of the last layer of the last residual block is selected as a source of feature maps, which are enriched to build a pyramid structure. Together with the feature map of the previous two blocks, upsampled by 2×, it completes the detection structure. This method changes the anchor boxes of the object detection model to make them suitable for face detection, which means that, anchor boxes, instead of having heights less than the widths, are built with narrow and tall boxes.

### 3.2. Preprocessing Stage

The preprocessing stage uses data techniques aimed at substantially improving the overall quality of features extracted in subsequent processes. There are different methods for data preprocessing and, depending on the problem to be solved and the data used for this purpose, it is necessary to satisfy the requirements of the intended use. Image preprocessing techniques can include color and geometric transformations, standardization, normalization, among others [56]. In the context of real-time facial recognition, the detected face images show size and illumination variations, and several typical preprocessing techniques must be applied such as image resampling and normalization. In general, when the acquired images have a non-homogeneous resolution and when the subsequent process images of the same size are necessary, resampling is used. Thus, for increasing the image resolution, the super resolution techniques are a suitable option available to transform the acquired image into an image with the desired resolution. This is necessary when deep learning models based on CNNs are used because these networks work with images of a unified dimension.

In this work, a CNN network [48] is used for feature extraction. This implies that the face image must be resampled to achieve a width and height identical to the images used for training the recognition system. For resampling the face images, a bicubic interpolation method was selected. Additionally, the L2 norm method is used, which is one of the most widely used normalization techniques in images. In this strategy, the data values are normalized based on a penalty of the sum of the squared absolute value because several studies demonstrate that the data normalization improves the performance of biometric systems [57,58,59]. This normalization technique has shown to improve the recognition accuracy, particularly under varying illumination [60].

**Figure 4:**
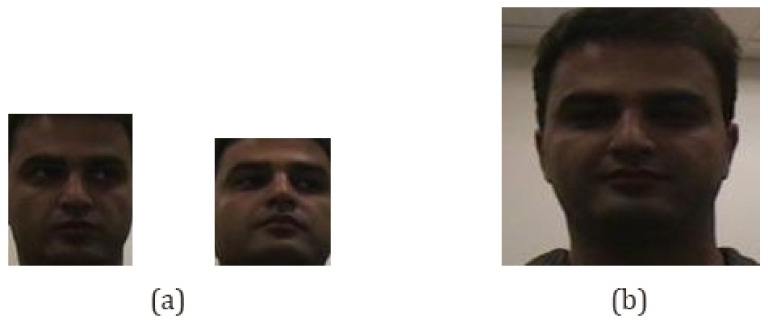
Image scaling problem. (**a**) face images obtained in the face detection stage and (**b**) image scale of the model training dataset.

The preprocessing techniques used in this work include color transformations, image scaling, and image normalization for the feature extraction methods. Color image conversion to grayscale is applied at the beginning of the face detection for the Viola–Jones method and image rescaling and image normalization are applied at the beginning of the feature extraction stage. Rescaling is performed because an image of the face on a different scale from the generalization of the model generates a non-conforming feature representation, as shown in Figure 4. The image normalization is performed to normalize the range of the pixel values of the input images; otherwise, each embed generated will have a high variation in the values.

### 3.3. Feature Extraction Stage

In recently proposed feature extraction methods, face image characteristics are estimated using a training scheme based on deep CNN, which are developed to be used in both the analysis and image face recognition. The main difference between an artificial neural network (ANN) and CNN is the fact that, while the conventional ANN consists of a fully connected input layer, one or more hidden layers, and an output layer, which perform a set of multiplications, additions, and nonlinear operation in each layer. On the other hand, the CNN consists of a set of hidden layers, consisting of a set of two-dimensional filters which are convolved with the input data of each layer. The outputs of these layers are fed into a set of pooling layers to reduce the amount of data in the next convolutional layer, and the output layers are one or two fully connected layers [61]. However, some current studies [11,12,14] challenge this rule.

Among the CNN-based schemes proposed for face recognition tasks, the *FaceNet* [48] is a well-known face recognition method, which is based on a CNN architecture that receives as input a set of face images and uses them to learn the image representation directly from the face images used for training. *FaceNet* is a deep neural network used to obtain facial features and determine if there is a matching between the input faces and a non-matching face with triplet-based loss function. Figure 1 presents the *FaceNet* structure, in which the face image is inserted into a deep CNN (Inception-ResNet [48]), which learns the features directly from the face image pixels, followed by an L2 normalization layer and finally get a 128-dimensional byte vector, which results in the face image represented by the face embedding. Lastly, FaceNet employs in the end-to-end learning the triplet loss function.

**Figure 5:**
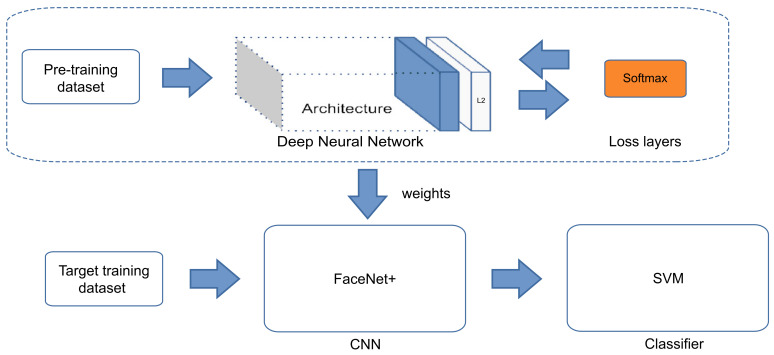
An overview of the proposed FaceNet+SVM framework.

**Figure 6:**
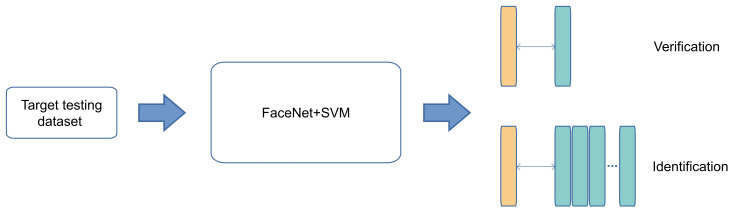
The testing of the proposed FaceNet+SVM framework.

Based on the idea of removing the fully connected layer in a CNN and plugging in a support vector machine (SVM) instead, we analyze the use of SVM, random forest (RF), and K-Nearest Neighbors (KNN), which are efficient supervised learning algorithms for classification [41,62,63] to create a combined architecture for each classifier for facial recognition task. Modifying the original FaceNet network, SVM, RF, or KNN are plugged instead of the last fully connected layer, followed by L2 normalization, implementing L2-SVM, L2-RF, and L2-KNN. The CNN weights are used as a baseline in this network, swapping the triplet loss function (proposed in FaceNet) for the softmax+cross-entropy loss function [64] in the training step, namely FaceNet+. Next, the weights are used to initialize the layers of CNN except its last layer. Subsequently, CNN is trained with the target training dataset to extract facial features and utilize these features to train the SVM. Figure 5 shows the structure of our proposed model and Figure 6 describes the recognition process of our system. We also analyze the models with KNN and RF instead of SVM. The proposed CNN+traditional classifier (such FaceNet+SVM, FaceNet+KNN and FaceNet+RF) architecture reduces the number of parameters through a majority of shared parameters, of about 65 K (sixty-five thousand) parameters. FaceNet+SVM is trained with C (regularization parameter) of $1\times 10^{3}$ and a linear kernel. FaceNet+KNN is trained using 10 neighbors, uniform weights, and Minkowski distance of 2. Finally, FaceNet+RF is trained using 100 trees in the forest and a minimum of 5 samples to be in a leaf node.

In recent years, the improvement of the network structure and the optimization of the loss function have been proposed to improve face classification and recognition such as VGGface, FaceNet, Cosface, and RingLoss. In this sense, the loss function used to model multiclass classification problems, Softmax+cross-entropy loss, has gained much attention and can be calculated as:

(1) $$L\left(y,\hat{y}\right)=-\frac{1}{N}\sum_{i=1}^{N}yi^{T}log\left(\hat{y}i\right)=-\frac{1}{N}\sum_{i=1}^{N}log\left(\hat{y}ig\right)$$

where *N* is the total number of samples to be identified $y_{i}\epsilon {\left\{\begin{array}{c} 0,1 \end{array}\right\}}^{j}$ is the label of the i-th sample in one-hot encoded representation, $\hat{y}i\epsilon {\left\{\begin{array}{c} 0,1 \end{array}\right\}}^{j}$ is the predicted probabilities with Softmax, and $\hat{y}ig$ is the predicted probability of the ground-truth class for the *i*-th sample.

This research and its contributions focus on adding a normalization layer and using the Sotfmax+cross-entropy loss function that can improve the final classification precision of FaceNet via L2-Softmax. Some improvements to the Softmax+cross-entropy loss function have been that the features learned by the convolutional neural network more distinctive between classes, that is, achieving a smaller intra-class distance and a larger inter-class distance. The experimental results show that our proposal improves the final precision in the LFW database and obtains a similar or higher score than different state-of-the-art CNN network structures compared to the existing loss functions.

## 4. Experimental Results

**Figure 7:**
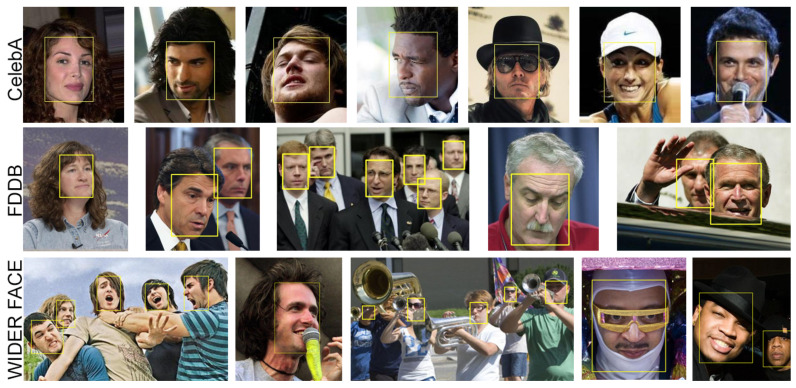
Example of a dataset used for face detection.

This section presents an extensive evaluation of face detection schemes, such as VJ, MTCNN, Face-SDD, and YOLOFace, to determine which is more suitable to be used in the proposed face recognition system. Firstly, extensive experiments are conducted on challenging face detection benchmarks: the Face Detection Data Set and Benchmark (FDDB) [65], CelebA dataset [66], WIDER FACE dataset [67] (see Figure 7), and Honda/UCSD dataset [68] to verify the effectiveness of YOLO Face. As for the face recognition stage, we verify the proposed models on two challenging benchmarks: Labeled Faces in the Wild (LFW) dataset [47] and YouTube Faces (YTF) dataset [69].

### 4.1. Datasets

In this subsection, we briefly describe the datasets used for evaluation of the proposed system.

FDDB. The FDDB dataset contains annotations for 5171 face images in a set of 2845 pictures, designed to study the problem of an unconstrained face detection. It includes a wide variety of challenges such as: low image resolutions, severe occlusions, and difficult poses.

CelebA. The CelebA dataset contains 202,599 face images of celebrities, each one with 40 annotations of attributes with large variations of pose and changes in the background.

WIDER FACE. The WIDER Face is a set of images for face detection with a high degree of facial expressions, occlusion, and illumination conditions. It contains 32,203 images and annotations of 393,703 faces. These images are split into three subsets: training (40%), validation (10%), and testing (50%) sets. Each subset contains three levels of difficulty: Easy, Medium, and Hard.

Honda/UCSD. The Honda/UCSD is a set of a standard video dataset used to evaluate the performance of face detection and tracking. This dataset offers a large amount of facial expressions and poses such as rotation in the 2D and 3D plane.

LFW. The LFW is a widely used dataset for evaluating face recognition systems, which contains more than 13,000 face images captured from the web of 1680 persons, with two or more different images per person.

YTF. The YouTube Faces is a video face dataset designed to study the unconstrained face recognition problem. This set contains 3425 videos of 1595 different persons.

### 4.2. Face Detection

The YOLO-Face, MTCNN, Face-SSD, and traditional methods are evaluated under the same conditions, using challenging datasets over our proposed system. Face detection methods are configured to find faces in input images with a possible facial object size of 20 px; facial objects smaller than that are ignored. To evaluate the performance of YOLO-Face and other detection methods, different metrics for evaluation of machine learning are used such as: precision, recall, accuracy, and average precision (AP). The metric employed in the dataset PASCAL VOC [70], IOU, an evaluation metric for the models developed for object localization, was also used. The IOU is defined as:

(2) $$IOU=B^{P}\cap B^{GT}/B^{P}\cup B^{GT}$$

where $B^{GT}$ =$\left(x^{GT},y^{GT},w^{GT},h^{GT}\right)$ is the face region and $B^{P}=\left(x,y,w,h\right)$ is the face detected region. Using Equation (1), a threshold was established as 0.5, such that, if the relation between the detected region and the labeled one is larger than the threshold, it is assumed that a face exists in the detected region; otherwise, it can be considered that a face in the detected region does not exist. The estimated values are expressed in percentage terms such that, when the system output approach to 100%, the face detection and face recognition accuracy improve.

#### 4.2.1. Main Results

YOLO-Face method was compared with other state-of-the-art methods [16,25,29] using FDDB, CelebA, and WIDER FACE. Although the YOLO-Face network has proven its accuracy in detecting faces, experimental results show some drawbacks with non-annotated faces. In such a way as to solve this problem, we propose a modification in the decision-making of the face detection stage. The proposal consists of an evaluation process to determine if the prediction was incorrect by comparing the confidence value of the YOLO-Face method. First, the comparison of the bounding box of the face and the annotated ground-truth is carried out, if it is determined that the bounding box is a false positive, it proceeds to a second comparison. Then, it is evaluated if the prediction is a false positive through the confidence value that YOLO-Face delivers; if the confidence value of the prediction is greater than or equal to 80%, it is determined that there is a face within the bounding box. Therefore, double comparison prevents unnoted faces from being evaluated as false positives.

**Figure 8:**
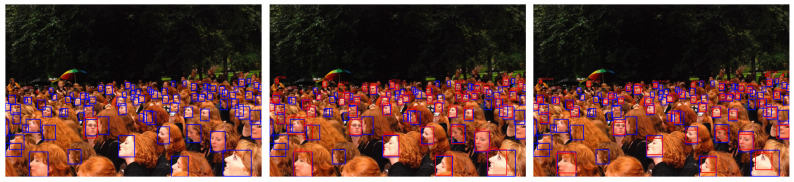
Some face detection result examples. (**Left**) results detected by Face-SSD; (**Middle**) results detected by MTCNN; (**Right**) results detected by YOLO-Face. The predicted location and the ground truth are in red and blue, respectively.

Table 1 shows the results obtained using the images contained in the FDDB dataset. Our experiments demonstrate that YOLO-Face provides a 99.8%, 72.9%, and 72.8% on precision, recall, and accuracy, only overcome by the MTCNN [25] with an accuracy of 81.5%. Additionally, the same face detection models were evaluated using the CelebA dataset which contains 19,962 image faces, whose detection results are shown in Table 2. Results show that, using the CelebA dataset, all the models obtain high performance. However, Face-SSD gets 99.7% accuracy over YOLO-Face with 99.6%. Finally, YOLO-Face obtains 95.8%, 94.2%, and 87.4% on the Easy, Medium, and Hard subsets of WIDER FACE validation are used, as highlighted in Table 3. Figure 8 shows some example face detection results on the WIDER dataset. We can easily see that the YOLO-Face detector performs better with small-scale faces.

We also evaluate YOLO-Face on a challenge video dataset [68] and compare it against some state-of-the-art methods [16,25,29]. Table 4 shows that the YOLO-Face method consistently outperforms all the previous approaches with the exception of video number 5 where Face-SSD has better performance.

#### 4.2.2. Face Detection Inference Time

Inference time was measured to demonstrate the efficiency of YOLO-Face compared with state-of-the-art methods using a single NVIDIA GTX 2080-TI with CUDA 10.2 and cuDNN 7.6.5. Frame-per-second (FPS) and milliseconds (ms) are used to compare the speed. As pictured in Table 5, the performance of YOLO-Face is better than the recent state of-the-art models with real-time speed. YOLO-Face spends 38 ms when detecting faces and can achieve about 26 fps for VGA-resolution (640 × 480) images. This method is 1.4 times faster than Refine Face [28] with better performance on the WIDER FACE validation and 9 ms faster than the other real-time method, Face-SSD.

Additionally, using an Intel(R) Core (TM) i7-7700HQ CPU, the measure of inference time of YOLO-Face was compared with state-of-the-art methods. Results are provided in Table 6. YOLO-Face can run at 2 FPS approximately using CPU, discarding it for CPU usage. Face-SSD achieves promising performance, and it can run at 24 FPS (50 ms) with an accuracy higher than 88% of detected faces on Honda/UCSD. In case the facial recognition application is limited by computational resources, the YOLO-Face used for face detection in the proposed scheme may be replaced by the Face-SSD scheme, with low performance degradation.

### 4.3. Face Recognition

At this stage, the baseline for the proposed methods is presented and provides the evaluation of the recognition performance. Our baseline is trained using the representations learned from CASIA-WebFace [71]. This training set consists of a total of 453,453 images of about 10 K (ten thousand) of different identities after face detection. Consequently, the adagrad optimizer was employed for an optimizer with learning rate of $10^{-3}$.

To measure the face recognition performance, proposed methods were evaluated for solving face recognition problems. Three different tasks of face recognition were considered: close-set face identification, open-set face identification, and face verification. In the tasks of face identification; three scenarios were defined. For each scenario, a training subset using the LFW dataset is defined, and Table 7 shows the training size for each of the scenarios. The methods for Scenario A were trained using 500 images of five different identities, the methods for Scenario B were trained using 960 images of 32 different identities, and the methods for Scenario C were trained using 1270 images of 127 identities.

The proposed methods FaceNet+SVM, FaceNet+KNN, and FaceNet+RF were compared against the baseline (FaceNet+) which are evaluated in scenarios A, B, and C for the face identification tasks. Table 8 shows the size of the testing subsets for each of the scenarios for the closed-set face identification. Table 9 shows the accuracy results of the evaluation in the three scenarios. In Scenario C, the evaluation results show that the FaceNet+SVM model was able to achieve an accuracy of 99.7% using the LFW dataset. On the other hand, the FaceNet+KNN and FaceNet+RF were able to achieve an accuracy of 99.5% and 89.1%, respectively, using the same scenario, whereas the FaceNet+ was able to achieve an accuracy of 99.6% with the same scenario.

**Figure 9:**
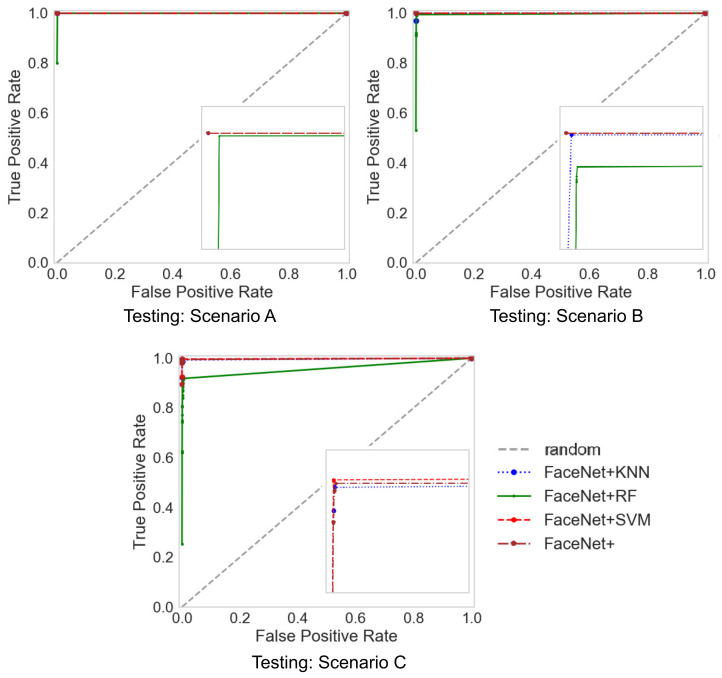
ROC curves in different scenarios of our proposed methods.

**Figure 10:**
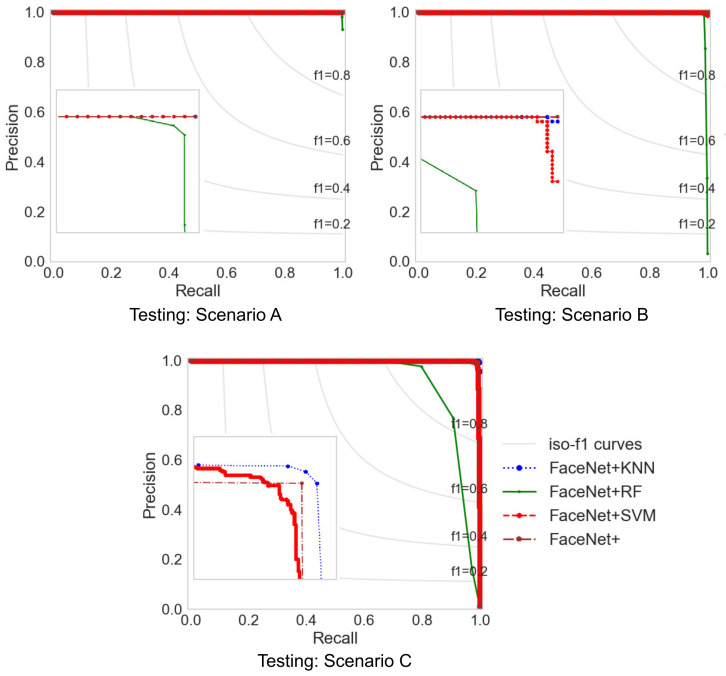
Precision–Recall curves in different scenarios of our proposed methods.

The evaluation results show that the proposed methods outperform the performance for identification even with a few images per person. The evaluation conditions are Area Under ROC Curve (AUC) and Average Precision (AP) are given in Table 10 with the SVM, k-Nearest Neighbor (KNN), Random Forest (RF) classifiers, and L2-Softmax loss. Each row in Table 10 corresponds to a compared facial recognition solution. Furthermore, Table 9 shows that, among the analyzed classifiers, the SVM provides better performance, followed by RF and finally KNN. Figure 9 and Figure 10 are the ROC (Receiver Operating Characteristic) and Precision–Recall comparison, respectively, for the proposed methods. In the identification task for the ROC curve, it is necessary to binarize the problem by using a one vs. all approach and calculating the operating points for each class (identity) and then perform micro or macro averaging of true positive (TP) and false negative (FN) ratios through the different classes, which measures the macro average of the proposed methods, giving equal weight to the classification of each class. Moreover, Precision–Recall calculates the trade-off between precision and recall using micro or macro averaging for each class through different thresholds. The macro average is used because the micro average might not represent the performance of our proposed methods at all classes.

Additionally, the best method, FaceNet+SVM, is evaluated for the task of open-set face identification on the LFW dataset with 12,733 images of 5749 different identities. The FaceNet+SVM is measured in terms of the accuracy with 5, 32, and 127 enrolled identities and different confidence scores (conf); the results on LFW are as listed in Table 11. Confidence scores are used to minimize the risk of mistakenly identifying the wrong person. Introducing confidence thresholds are important in the open-set contexts where the probes include non-enrolled identities.

**Figure 11:**
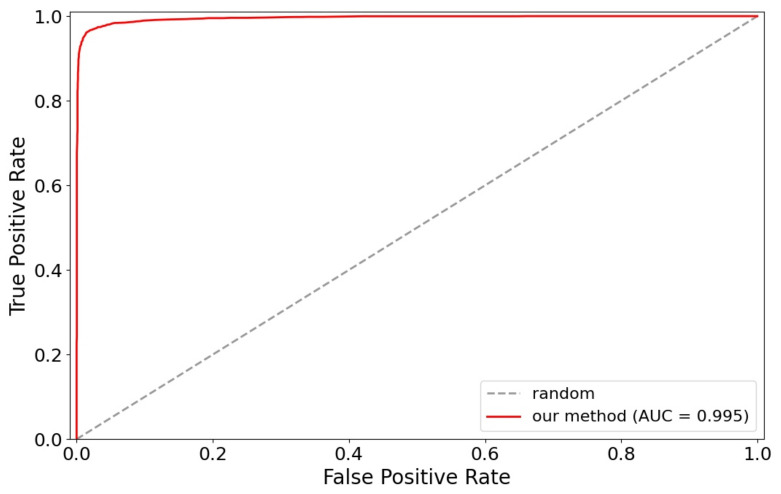
ROC curve for verification tasks on LFW.

The proposed FaceNet+SVM method on the face verification task according to the experiment presented by Schroff et al. is also evaluated [48] for independent subjects. Given a pair of face images, the Euclidean distance is computed to determine if the images are the same or different. In addition, 99.7% and 94.7% classification accuracy were achieved on LFW and YTF datasets, respectively. Table 12 shows the accuracy as compared with the previously mentioned state-of-the-art approaches on the most popular LFW benchmark for face verification tasks. These results show that our FaceNet+SVM relatively improves FaceNet [48] and diverse solutions such DeepID3 [49], Cosface [51], and Ring loss [52]. In the same way, Table 13 shows the accuracy of different methods evaluated on another challenging dataset, YTF. Table 14 and Table 15 show the accuracy of some methods trained with the same database. In addition, Figure 11 shows the ROC curve with our FaceNet+SVM method for verification tasks on the LFW dataset.

During testing, the face recognition stage spends 12 ms for an VGA image on the GPU, of which 11.5 ms are required for preprocessing and feature extraction, and 0.5 ms is required for face matching using our proposed method, FaceNet+SVM. In the proposed system, when combining face detection and face recognition stages, 99.1% recognition accuracy was obtained on the YTF dataset and real-time speed of 24 FPS at GPU using the YOLO-Face method and the FaceNet+SVM method. Additionally, the identification rate is evaluated, with the number of faces correctly identified (true positive rate) and the number of faces that are not correctly identified (false negative rate), using videos of the YTF dataset and YOLO-Face method and the FaceNet+SVM method; the results are shown in Table 16. These results show that, in a closed set environment, when the face detection is wrong, that is, it detects a fake face or a bounding box that contains an object that is not really a face, the recognition rate worsens. For example, in videos 3, 7 and even in video 10, the detection system clearly fails to correctly detect the faces contained in the videos and then, in such situations, the achieved recognition or verification rates become too low. This limitation of the proposed system can be solved if some confidence thresholds are inserted, which are explained in Table 16.

#### Complete System Inference Time

The inference time of the complete system was measured with the proposed pipeline by comparing the methods proposed in the detection stage and one of the best models in the recognition stage, FaceNet+SVM, using a single NVIDIA GTX 2080-TI with CUDA 10.2 and cuDNN 7.6.5. Frame-per-second (FPS) and millisecond (ms) are used to compare the speed; the results are provided in Table 17. The results show a shorter execution time using the YOLO-Face and FaceNet+SVM methods; our framework can run at 24 FPS (49 ms) with the possibility of being used in real-time applications.

## 5. Conclusions

This paper proposed a general scheme for implementing a facial recognition system in a real-time video. To this end, several different solutions, using machine learning and deep learning approaches, were analyzed in a systematic form. From this analysis, more suitable schemes for face image detection, feature extraction, and classification tasks were selected. YOLO-Face, a one-stage deep learning detector, was compared with several state-of-the-art face detection algorithms, including handcrafted-based as well as deep learning-based approaches. These schemes were evaluated operating in unconstrained environments using images as well as video dataset to determine its performance, computational complexity, as well as if they are suitable for implementation in a real-time environment. Experimental results demonstrate that, when a GPU is available, the YOLO-Face scheme provides better recognition results with faster processing speed, while, when only a CPU is available, the Face-SSD provides the best compromise between computation speed and recognition rate. In this situation, the Face-SSD requires only 50 ms for recognition while its performance is very close to the detection rate provided by the YOLO-Face. Then, when a computer system with limited capacity is available, the YOLO-Face used for face detection in the proposed scheme may be replaced by the Face-SSD scheme, with low performance degradation. The handcrafted methods of VJ, LBP, and HOG present reasonably good performance in many practical situations, although their performance degrades in highly complex scenarios. In general, the YOLO-Face provides a better performance even in environments with large changes in illumination, pose variations, occlusion, etc. Although MTCNN shows better performance, it is 25% slower using a GPU. On the other hand, the proposed scheme which combines FaceNet+SVM is highly accurate for identifying previously learned persons by using face characteristics in unconstrained environments. Quantitative results show that an architecture combining FaceNet+SVM or FaceNet+KNN has similar performance to FaceNet [48]. In summary, with the advances of deep learning algorithms together with the technological advances, it is possible to develop real-time face recognition systems capable of operating in highly complex environments. Future work will evaluate the proposed system with other databases and compare them with some recent state-of-the-art systems.

## Figures and Tables

**Table 1:** Detection results on the FDDB dataset.

	Precision (%)	Recall (%)	Accuracy (%)
VJ [16]	**99.9**	50.0	50.1
MTCNN [25]	95.1	**85.1**	**81.5**
Face-SSD [29]	98.8	33.9	33.8
YOLO-Face [31]	99.8	72.9	72.8

**Table 2:** Detection results on the CelebA dataset.

	Precision (%)	Recall (%)	Accuracy (%)
VJ [16]	99.9	89.1	92.2
MTCNN [25]	98.7	99.0	98.6
Face-SSD [29]	99.9	**99.7**	**99.7**
YOLO-Face [31]	**99.9**	99.6	99.6

**Table 3:** Detection results on the WIDER FACE validation subset, evaluated with AP (%) on the validation subset.

	Easy (AP%)	Medium (AP%)	Hard (AP%)
VJ [16]	41.2	33.3	13.7
MTCNN [25]	84.8	82.5	59.8
Face-SSD [29]	92.1	89.5	71.6
YOLO-Face [31]	**95.8**	**94.2**	**87.4**

**Table 4:** Accuracy of the different face detection models on the Honda/UCSD video dataset.

Videos	Num. of Frame	Detection Rate (%)			
		VJ [16]	MTCNN [25]	Face-SSD [29]	YOLO-Face [31]
1	387	76.3	94.0	96.8	**97.3**
2	313	61.7	91.7	99.0	**99.6**
3	394	48.6	82.2	93.4	**95.1**
4	474	49.6	82.1	95.3	**96.0**
5	368	44.2	84.2	**95.3**	94.5
6	352	42.6	92.4	94.0	**96.3**
7	290	47.0	84.1	97.5	**99.9**
8	310	39.5	80.3	88.1	**89.6**
9	663	77.3	99.2	99.5	**99.9**
10	418	58.7	90.9	94.9	**95.4**

**Table 5:** Time comparison between different face detection methods for VGA images on NVIDIA GTX 2080-TI GPU.

	Backbone	Time	FPS	GPU Type
VJ [16]	-	41 ms	24	2080TI
MTCNN [25]	PNet-RNet-ONet	66 ms	18	2080TI
Face-SSD [29]	VGG-16	47 ms	24	2080TI
YOLO-Face [31]	Darknet-53	38 ms	26	2080TI

**Table 6:** Time comparison between different face detection methods for VGA images on CPU.

	Backbone	Time	FPS	CPU Type
VJ [16]	-	41 ms	24	i7-7700HQ
MTCNN [25]	PNet-RNet-ONet	110 ms	8	i7-7700HQ
Face-SSD [29]	VGG-16	50 ms	24	i7-7700HQ
YOLO-Face [31]	Darknet-53	430 ms	2	i7-7700HQ

**Table 7:** Size of training subsets on LFW for face recognition.

	No. of Images	No. of Identities	No. of Images per Identity
Scenario A	500	5	100
Scenario B	960	32	30
Scenario C	1270	127	10

**Table 8:** Size of testing subsets on LFW for face recognition.

	No. of Images	No. of Identities
Scenario A	640	5
Scenario B	1350	32
Scenario C	2728	127

**Table 9:** Close-set face recognition accuracy on testing scenarios A, B, and C.

	Scenario A	Scenario B	Scenario C
FaceNet+	100.0%	99.0%	99.6%
Proposed FaceNet+SVM	99.9%	99.9%	99.7%
Proposed FaceNet+KNN	99.9%	99.9%	99.5%
Proposed FaceNet+RF	99.9%	99.0%	89.1%

**Table 10:** AUC and AP comparison in different scenarios of our proposed methods.

Method	Scenario A		Scenario B		Scenario C	
	AUC	AP	AUC	AP	AUC	AP
FaceNet+SVM	99.99%	99.99%	99.99%	99.99%	99.81%	99.73%
FaceNet+KNN	99.99%	99.99%	99.98%	99.99%	99.69	99.95%
FaceNet+RF	99.96%	99.98%	99.68%	99.32%	95.87	88.98%
FaceNet+	99.99%	99.99%	99.99%	99.99%	99.75	98.76%

**Table 11:** Open-set face recognition accuracy with the FaceNet+SVM on LFW.

	Conf = 0.6	Conf = 0.7	Conf = 0.8	Conf = 0.9
5 identities	72.2%	86.2%	94.7%	98.8%
32 identities	89.4%	89.0%	89.0%	89.0%
127 identities	76.7%	76.7%	76.7%	76.7%

**Table 12:** Face recognition of different methods on the LFW dataset.

Method	Loss	Training Set	Accuracy (%)
DeepFace [45]	softmax	Facebook (4.4 M, 4 K)	97.3
DeepFace+ [71]	contrastive loss	CASIA-WebFace (0.4 M, 10 K)	97.7
DeepID2 [72]	contrastive loss	CelebFaces+ (0.2 M, 10 K)	99.1
DeepID2+ [46]	contrastive loss	CelebFaces+ & WDRef (0.2 M, 12 K)	99.4
DeepID3 [49]	contrastive loss	CelebFaces+ (0.2 M, 10 K)	99.5
VGGface [73]	triplet loss	VGGface (2.6 M, 2.6 K)	98.9
FaceNet [48]	triplet loss	Google (500 M, 10 M)	99.6
L2-softmax [50]	L2-softmax	MS-Celeb-1 M (3.7 M, 58 K)	99.7
Cosface [51]	cosface	CASIA-WebFace (0.4 M, 10 K)	99.3
Ring loss [52]	Ring loss	MS-Celeb-1 M (3.5 M, 31 K)	99.5
Arcface [53]	arcface	MS-Celeb-1 M (3.7 M, 58 K)	**99.8**
FaceFilter [54]	triplet+Kullback–Leibler loss	CelebFaces+ (0.2 M, 10 K)	99.7
Our method	L2-softmax	CASIA-WebFace (0.4 M, 10 K)	99.7

**Table 13:** Face recognition of different methods on the YTF dataset.

Method	Loss	Training Set	Accuracy (%)
DeepFace [45]	softmax	Facebook (4.4 M, 4 K)	90.4
DeepFace+ [71]	contrastive loss	CASIA-WebFace (0.4 M, 10 K)	92.2
DeepID2+ [46]	contrastive loss	CelebFaces+ & WDRef (0.2 M, 12 K)	93.2
VGGFace [73]	triplet loss	VGGface (2.6 M, 2.6 K)	**97.3**
FaceNet [48]	triplet loss	Google (500 M, 10 M)	95.1
FaceFilter [54]	triplet+Kullback–Leibler loss	CelebFaces+ (0.2 M, 10 K)	94.0
Our method	L2-softmax	CASIA-WebFace (0.4 M, 10 K)	94.7

**Table 14:** Face recognition of different methods on the LFW dataset (using the same training dataset).

Method	Loss	Training Set	Accuracy (%)
DeepFace+ [71]	contrastive loss	CASIA-WebFace (0.4 M, 10 K)	97.7
Cosface [51]	cosface	CASIA-WebFace (0.4 M, 10 K)	99.3
Our method	softmax+cross-entropy loss	CASIA-WebFace (0.4 M, 10 K)	**99.7**

**Table 15:** Face recognition of different methods on the YTF dataset (using the same training dataset).

Method	Loss	Training Set	Accuracy (%)
DeepFace+ [71]	contrastive loss	CASIA-WebFace (0.4 M, 10 K)	92.2
Our method	softmax+cross-entropy loss	CASIA-WebFace (0.4 M, 10 K)	**94.7**

**Table 16:** Identification rate with our proposed system evaluated on the YTF dataset. The YOLO-Face model and the FaceNet+SVM model are adopted for face detection and face recognition, respectively.

Videos	# Frames	Detection Rate (%)	Identification Rate (%)	End-to-End Recognition Rate (%)
1	106	90.6	99.9	90.5
2	77	99.9	99.9	99.9
3	65	23.1	0.0	0.0
4	57	99.9	98.2	98.1
5	128	99.9	97.6	97.5
6	69	99.9	99.9	99.9
7	365	69.6	5.9	4.10
8	201	99.9	99.9	99.9
9	61	95.1	99.9	95.0
10	96	84.4	14.8	12.5

**Table 17:** Performance of face recognition systems in unconstrained environments.

	Identification Rate	Time	FPS	GPU Type
VJ & FaceNet+SVM	79.10%	53 ms	23	2080TI
MTCNN & FaceNet+SVM	87.50%	75 ms	13	2080TI
Face-SSD & FaceNet+SVM	94.80%	60 ms	19	2080TI
YOLO-Face & FaceNet+SVM	95.87%	49 ms	24	2080TI

## Data Availability

Not applicable.
